# Indocyanine green fluorescence navigation in laparoscopic hepatectomy: a retrospective single-center study of 120 cases

**DOI:** 10.1007/s00595-020-02163-8

**Published:** 2020-10-31

**Authors:** Hao Lu, Jian Gu, Xiao-feng Qian, Xin-zheng Dai

**Affiliations:** Hepatobiliary Center, First Affiliated Hospital, Nanjing Medical University, 300 Guangzhou Road, Nanjing, 210029 Jiangsu China

**Keywords:** Laparoscopy, Hepatectomy, Indocyanine green, Navigation

## Abstract

**Purpose:**

To explore the role of indocyanine green (ICG) fluorescence navigation in laparoscopic hepatectomy and investigate if the timing of its administration influences the intraoperative observation.

**Methods:**

The subjects of this retrospective study were 120 patients who underwent laparoscopic hepatectomy; divided into an ICG-FN group (*n* = 57) and a non-ICG-FN group (*n* = 63). We analyzed the baseline data and operative data.

**Results:**

There were no remarkable differences in baseline data such as demographic characteristics, lesion-related characteristics, and liver function parameters between the groups. Operative time and intraoperative blood loss were significantly lower in the ICG-FN group. The rate of R0 resection of malignant tumors was comparable in the ICG-FN and non-ICG-FN groups, but the wide surgical margin rate was significantly higher in the ICG-FN group. The administration of ICG 0–3 or 4–7 days preoperatively did not affect the intraoperative fluorescence imaging. Operative time, intraoperative blood loss, and a wide surgical margin correlated with ICG fluorescence navigation. ICG fluorescence navigation helped to minimize intraoperative blood loss and achieve a wide surgical margin.

**Conclusion:**

ICG fluorescence navigation is safe and efficient in laparoscopic hepatectomy. It helps to achieve a wide surgical margin, which could result in a better prognosis. The administration of ICG 0–3 days preoperatively is acceptable.

**Electronic supplementary material:**

The online version of this article (10.1007/s00595-020-02163-8) contains supplementary material, which is available to authorized users.

## Introduction

Laparoscopic hepatectomy is now performed widely to cure benign and malignant liver diseases [1]. To mark the demarcation line, portal staining or inflow clamping of the target area is recommended in conventional open anatomical liver resection [2–4]. Conversely, in laparoscopic hepatectomy, this requires advanced skills. The lack of tactile perception of laparoscopic forceps and the complexity of intraoperative ultrasound examination makes it challenging to localize the tumor and confirm the demarcation line, limiting the application of this technology [5].

Indocyanine green (ICG), once bound to protein, can emit fluorescence (peaking at 840 nm) under the illumination of near-infrared light [6]. Because it can be absorbed exclusively by hepatocytes and excreted through bile without enterohepatic recirculation, ICG has gain the attention of hepatobiliary surgeons over the last two decades [7]. Intraoperative ICG fluorescence navigation might be a complementary solution to overcome the limitation of laparoscopic hepatectomy [4, 8]. Its application can also improve the detection rate of liver tumor focus, especially small lesions in an early stage [9–11]. Moreover, laparoscopic hepatectomy using ICG fluorescence navigation is associated with less intraoperative blood loss, a lower transfusion rate, less postoperative complications, and reduced hospital stay, as well as a higher R0 resection rate to avoid a positive surgical margin [12–15].

Despite all these advantages, ICG fluorescence navigation has some inherent drawbacks. First, ICG is usually injected intravenously a few days prior to the operation and bile metabolism malfunctions in diseased (especially cirrhotic) liver, and residual ICG will affect the observation [13, 16]. Second, regenerated nodules might show as false-positive if the bile duct is compressed [9]. Third, ICG fluorescence can only penetrate about 5–10 mm of tissue, so deep tumors cannot be detected superficially and positive- and negative-staining must be combined. More studies are needed to demonstrate the importance of ICG fluorescence navigation in laparoscopic hepatectomy.

We conducted this retrospective study, based on our 120-patient, single-center experience of laparoscopic hepatectomy, to investigate the role of ICG fluorescence navigation in laparoscopic hepatectomy and to assess the influence of the timing of ICG administration on intraoperative observation.

## Methods

### Patients and grouping

All patients who underwent laparoscopic hepatectomy between January, 2018 and December, 2019, were included in this retrospective study, with the exclusion of those under18 years of age, those with a history of prior upper abdominal surgery, and those who required conversion to open surgery. All operations were performed by the same team of surgeons who had completed the necessary training. The120 patients enrolled were divided into an ICG-FN group and a non-ICG-FN group, according to the availability of the ICG-FN imaging system. The ICG-FN group was divided further into a 0–3 days subgroup and a 4–7 days subgroup according to number of days preoperatively that ICG was administered. This retrospective study was approved by the Institutional Review Board of the First Affiliated Hospital, Nanjing Medical University (No. 2020-SR-124).

### Surgical procedure and ICG fluorescence navigation

To obtain R0 resection and preserve maximal liver parenchyma, non-anatomic or anatomic resection was selected flexibly, depending on lesion-related characteristics and the preoperative liver function of the patient. There was no difference in the surgical procedure between the groups. The PINPOINT PC9000 (NOVADAQ, Canada) endoscopic system was used for fluorescence navigation. ICG was injected intravenously (0.5 mg/kg body weight) into a peripheral vein, 0–7 days before or during the operation, for tumor-specific or negative staining, respectively.

### Observation indexes

The demographic characteristics (gender and age), lesion-related characteristics (lesion pathology type, number of involved lobes, and diameter of measurable lesion), and liver function parameters [degree of cirrhosis, volume of ascites, and ICG R15 (ICG retention rate after 15 min)] were obtained as baseline data in all patients. Observation indexes included the operative method (anatomical/non-anatomical resection), operative time, hilar occlusion time, intraoperative blood loss, intraoperative transfusion, postoperative complications, and postoperative hospital stay for all patients, as well as pathological evaluation of the surgical margin for malignancy in both groups. In the ICG-FN group, intraoperative imaging was assessed by both the surgeon and the assistant, who checked for clear staining in the target area, with visible boundaries and no residual ICG or diffusion in the non-target area.

### Statistical analysis

Statistical analysis was performed using SPSS 22.0 software. The mean and standard deviation (SD) were calculated for normally distributed variables, with median and the 1st/3rd quartiles for skewed data. The Pearson *χ*^2^ test was used to compare differences in frequencies. A continuous corrected *χ*^2^ test was used if the theoretical value was between 1 and 5, and the Fisher-exact test was used if it was less than 1. Student's *t* test and Mann–Whitney *U* test were used to compare mean and median values between the groups. Spearman's rank correlation analysis was used to analyze the correlation between treatment measures and clinical observation indexes. Backward stepwise logistic regression models were used to assess the association between variables and clinical observations indexes (continuous variables were converted to categorical variables), after adjustment for ICG fluorescence navigation, gender, age, number of involved lobes, diameter of measurable lesion, degree of cirrhosis, volume of ascites, ICG R15, and operation method. A value of *p* < 0.05 was considered significant.

## Results

### Baseline characteristics in the ICG-FN and non-ICG-FN groups

There were 57 patients in the ICG-FN group and 63 patients in the non-ICG-FN group. There were no significant differences in the demographic characteristics (gender and age), lesion-related characteristics (pathology type, number of involved lobes, and diameter of measurable lesion), and liver function parameters (degree of cirrhosis, volume of ascites, and ICG R15) between the ICG-FN and non-ICG-FN groups (*p* > 0.05 for all; Table 1).

**Table 1:** Baseline characteristics of the patients in both groups

	ICG-FN (*n* = 57)	non-ICG-FN (*n* = 63)	*p* value
Demographics			
Gender (*n*, %)			0.587^a^
Male	38 (66.7)	39 (61.9)	
Female	19 (33.3)	24 (38.1)	
Age (mean ± SD)	57.3 ± 12.2	55.2 ± 12.5	0.341^b^
Lesion			
Pathology type (*n*, %)			0.455^a^
Malignant	39 (68.4)	39 (61.9)	
HCC	28	30	
ICC	6	4	
HCC/ICC mixed	1	2	
Other primary liver cancer	1	0	
Metastatic liver cancer	3	3	
Benign	18 (31.6)	24 (61.9)	
Hemangioma	5	15	
PEComa	2	0	
FNH	4	0	
Hepatolithiasis	3	3	
Inflammation and necrosis	4	7	
Number of involved lobes (*n*, %)			0.969^a^
Single	49 (86)	54 (85.7)	
Multiple	8 (14)	9 (14.3)	
Diameter of measurable lesion [cm, median (1st/3rd quartiles, *n*)]	4.5 (2.5–5.1, 54)	4.5 (3.2–6.5, 60)	0.263^c^
Liver function			
Degree of cirrhosis (*n*, %)			0.235^a^
≦Mild	43 (75.4)	53 (84.1)	
≧Moderate	14 (24.6)	10 (15.9)	
Volume of ascites (*n*, %)			0.313^a^
None	47 (82.5)	56 (88.9)	
Mild	10 (17.5)	7 (11.1)	
ICG R15 [median (1st/3rd quartiles)]	4.2 (2.1–7.4)	3.7 (2.4–6.7)	0.801 ^c^

*ICG* indocyanine green, *FN* fluorescence navigation, *SD* standard deviation, *HCC* hepatocellular carcinoma, *ICC* intrahepatic cholangiocarcinoma, *PEComa* perivascular epithelioid cell tumor, *FNH* focal nodular hyperplasia, *ICG R15* ICG retention rate after 15 min

^a^Pearson *χ*^2^ test

^b^Student’s *T* test

^c^Mann–Whitney *U* test

### Operation and recovery indexes in the ICG-FN and non-ICG-FN groups

The operative time and intraoperative blood loss were significantly lower in the ICG-FN group than in the non ICG-FN group, being 160 (115–195) min vs. 180 (125–225) min (*p* = 0.035) and 100 (35–200) ml vs. 200 (100–400) ml (*p* = 0.025), respectively. The operation method (anatomical/non-anatomical resection), hilar occlusion time, intraoperative transfusion, incidence of postoperative complications, and postoperative hospital stay did not differ significantly between the groups (Table 2).

**Table 2:** Operation and recovery indices in both groups

	ICG-FN (*n* = 57)	non-ICG-FN (*n* = 63)	*p* value
Operation			
Method (*n*, %)			0.800^a^
Anatomical resection	23 (40.4)	24 (38.1)	
Non-anatomical resection	34 (59.6)	39 (61.9)	
Operative time [min, median (1st/3rd quartiles)]	160 (115–195)	180 (125–225)	0.035^b^
Hilar occlusion time [min, median (1st/3rd quartiles)]	20 (0–47)	21 (0–51)	0.883^b^
Intraoperative blood loss [ml, median (1st/3rd quartiles)]	100 (35–200)	200 (100–400)	0.025^b^
Intraoperative transfusion (*n*, %)			0.623^a^
Yes	6 (10.5)	5 (7.9)	
No	51 (89.5)	58 (92.1)	
Recovery			
Postoperative complication (*n*, %)			0.711^a^
Yes	6 (10.5)	8 (12.7)	
No	51 (89.5)	55 (87.3)	
Postoperative hospital stay [day, median (1st/3rd quartiles)]	7 (6–9)	8 (7–10.5)	0.183^b^

^a^Pearson *χ*^2^ test

^b^Mann–Whitney *U* test

### Pathological evaluation of the surgical margin for malignant tumors

Overall, there were 39 patients who underwent surgery for malignant disease, accounting for 68.4% and 61.9% of the total population in each group, with no difference in composition ratio (Table 1). Pathological evaluation of the surgical margin (Table 3) suggested that the R0 resection rate was comparable in the ICG-FN and non-ICG-FN groups (100.0 vs. 94.9%, *p* = 0.474). Therefore, we further compared the cases of a wide surgical margin (margin width > 10 mm) in both groups and found the rate of a wide surgical margin to be significantly higher in the ICG-FN group than in the non-ICG-FN group (92.3 vs. 74.4%, *p* = 0.033).

**Table 3:** Pathological evaluation of the surgical margin in malignant tumor resection in both groups

	ICG-FN (*n* = 39)	non-ICG-FN (*n* = 39)	*p *value
R0 resection vs. R1 resection (*n*, %)			0.474^b^
R0	39 (100.0%)	37 (94.9%)	
R1	0 (0.0%)	2 (5.1%)	
Wide margin vs. narrow margin (*n*, %)			0.033^a^
Wide (> 10 mm)	36 (92.3%)	29 (74.4%)	
Narrow (≤ 10 mm)	3 (7.7%)	10 (25.6%)	

^a^Pearson *χ*^2^ test

^b^Continuous corrected *χ*^2^ test

### Intraoperative fluorescence imaging satisfaction analysis

Fluorescence imaging satisfaction was evaluated intraoperatively in the ICG-FN group by both the surgeon and the assistant. The imaging quality was not clear enough to proceed with surgery in 3 (7.5%) of the 40 patients in the 0–3 preoperative days subgroup, but no significance was indicated (*p* = 0.547; Table 4). The operative time, hilar occlusion time, intraoperative blood loss, and postoperative hospital stay were all comparable (*p* > 0.05 for all).

**Table 4:** Effects of preoperative timing of indocyanine green administration on intraoperative fluorescence imaging satisfaction assessment and other indices

	0–3 day (*n* = 40)	4–7 day (*n* = 17)	*p* value
Satisfaction analysis (yes/no) (*n*, %)	37 (92.5)/3 (7.5)	17 (100)/ 0 (0)	0.547^b^
Operative time [min, median (1st/3rd quartiles)]	155 (110–195)	165 (132.5–207.5)	0.246^a^
Hilar occlusion time [min, median (1st/3rd quartiles)]	17 (0–43.75)	27 (0–49)	0.541^a^
Intraoperative blood loss [ml, median (1st/3rd quartiles)]	100 (50–200)	150 (20–275)	0.798^a^
Postoperative hospital stay [day, median (1st/3rd quartiles)]	8 (6.25–10.75)	9 (7–10.5)	0.342^a^

^a^Mann–Whitney *U* test

^b^Fisher exact test

### Spearman's rank correlation and backward stepwise logistic regression analysis of operative time and intraoperative blood loss for all patients

Spearman's rank correlation analysis revealed that the operative time (*r* = − 0.193, *p* = 0.035) and intraoperative blood loss (*r* = − 0.205, *p* = 0.025) were negatively correlated with ICG fluorescence navigation (Table 5), but not with gender, age, lesion pathology type, number of involved lobes, diameter of measurable lesion, degree of cirrhosis, volume of ascites, ICG R15, and hepatectomy method. Backward stepwise logistic regression analysis further indicated that the risk factor for operative time was the diameter of a measurable lesion (OR 1.221, 95% CI 1.052–1.419, *p* = 0.009), the risk factors for intraoperative blood loss were the diameter of a measurable lesion (OR 1.183, 95% CI 1.021–1.370, *p* = 0.025) and age (OR 1.035, 95% CI 1.003–1.069, *p* = 0.032), and that ICG fluorescence navigation was a protective factor (OR 0.446, 95% CI 0.205–0.967, *p* = 0.041) (Table 6).

**Table 5:** Spearman's rank correlation analysis of operative time, intraoperative blood loss, and baseline indices in all patients

	Operation time	Intraoperative blood loss
ICG-FN		
* r*	−0.193	−0.205
* p*	0.035	0.025

**Table 6:** Backward stepwise logistic regression analysis of operative time and intraoperative blood loss in all patients

Variables	Operation time			Intraoperative blood loss		
	OR	95% CI	*p*	OR	95% CI	*p*
ICG-FN				0.446	0.205–0.967	0.041
Diameter of measurable lesion	1.221	1.052–1.419	0.009	1.183	1.021–1.370	0.025
Age				1.035	1.003–1.069	0.032

*OR* odds ration, *CI* confidence interval

### Spearman's rank correlation and backward stepwise logistic regression analysis of wide surgical margin for malignant tumors

Spearman's rank correlation analysis suggested a positive correlation between a wide surgical margin and ICG fluorescence navigation (*r* = 0.241, *p* = 0.034; Table 7), but not to gender, age, lesion pathology type, number of involved lobes, diameter of measurable lesion, degree of cirrhosis, volume of ascites, ICG R15, or hepatectomy method. Backward stepwise logistic regression analysis illustrated that ICG fluorescence navigation was the only protective factor (OR 4.138, 95% CI 1.041–16.444, *p* = 0.044; Table 7) to obtain a wide surgical margin.

**Table 7:** Spearman's rank correlation and backward stepwise logistic regression analysis of a wide margin in resection of malignant tumors

Variables	Spearman's rank correlation		Backward stepwise logistic regression		
	*r*	*p*	OR	95%CI	*p*
ICG-FN	0.241	0.034	4.138	1.041–16.444	0.044

### Effects of liver resection type on operation and recovery of all patients and the surgical margin for malignant tumors

There were no significant differences in operative time, hilar occlusion time, intraoperative blood loss, postoperative hospital stay, and wide or negative surgical margin rate between patients who underwent anatomical vs. those who underwent non-anatomical liver resection (Tables S1 and S2).

## Discussion

Recent studies have demonstrated the safety and efficiency of ICG fluorescence navigation in laparoscopic hepatectomy [12–15]. This study also showed that the intraoperative and postoperative indexes of the ICG-FN group were comparable to or even better than those of the non-ICG-FN group. For patients with malignant tumors, the residual ICG inside the lesion may emit fluorescence under near-infrared light illumination, which helps the surgeon find the lesion quickly, especially if the tumor is in an “inconvenient” location such as the right posterior lobe [10, 11, 17]. The implementation of a negative staining technique is also helpful to identify the liver segment/lobe boundary and the resection plane. Energy devices such as the ultracision harmonic scalpel (Johnson & Johnson) are capable of breaking the liver parenchyma in the non-vascular area between the liver segments, reducing the need for vascular clips, reducing the operative time, preventing large vessel injury in the liver segments, and minimizing intraoperative blood loss. This was confirmed in our study. As a result of advances in surgical techniques, the overall intraoperative transfusion rate was low, with no difference between the groups. Although studies have shown that fluorescence navigation can reduce the incidence of bile leakage and liver abscess after hepatic segmentectomy and lobectomy [12], and minimize the postoperative hospital stay [13, 14], this study did not find that ICG fluorescence navigation reduced postoperative complications or the postoperative hospital stay. However, Spearman’s rank correlation and stepwise Logistic regression analysis indicated that ICG fluorescence navigation was associated with shorter operative time and less intraoperative blood loss. It was also a protective factor for less intraoperative blood loss, proving its safety and effectiveness. In this study, the liver resection type (anatomical vs. non-anatomical) did not affect operation time, hilar occlusion time, intraoperative blood loss, or postoperative hospital stay.

**Fig. 1.**
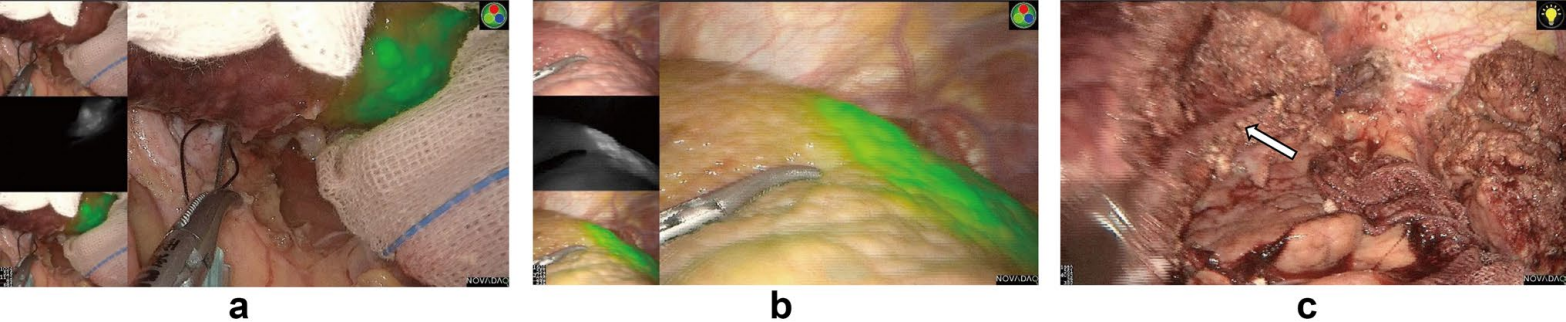
ICG was injected into segment 2 portal branch (black arrow) **a**. Segment 2 was stained immediately and a superficial tumor near the liver capsular was visualized on the fluorescence fusion image under illumination of near infrared light **b**. This segment was resected using ICG fluorescence navigation and left hepatic vein was indicated (white arrow) **c**

**Fig. 2.**
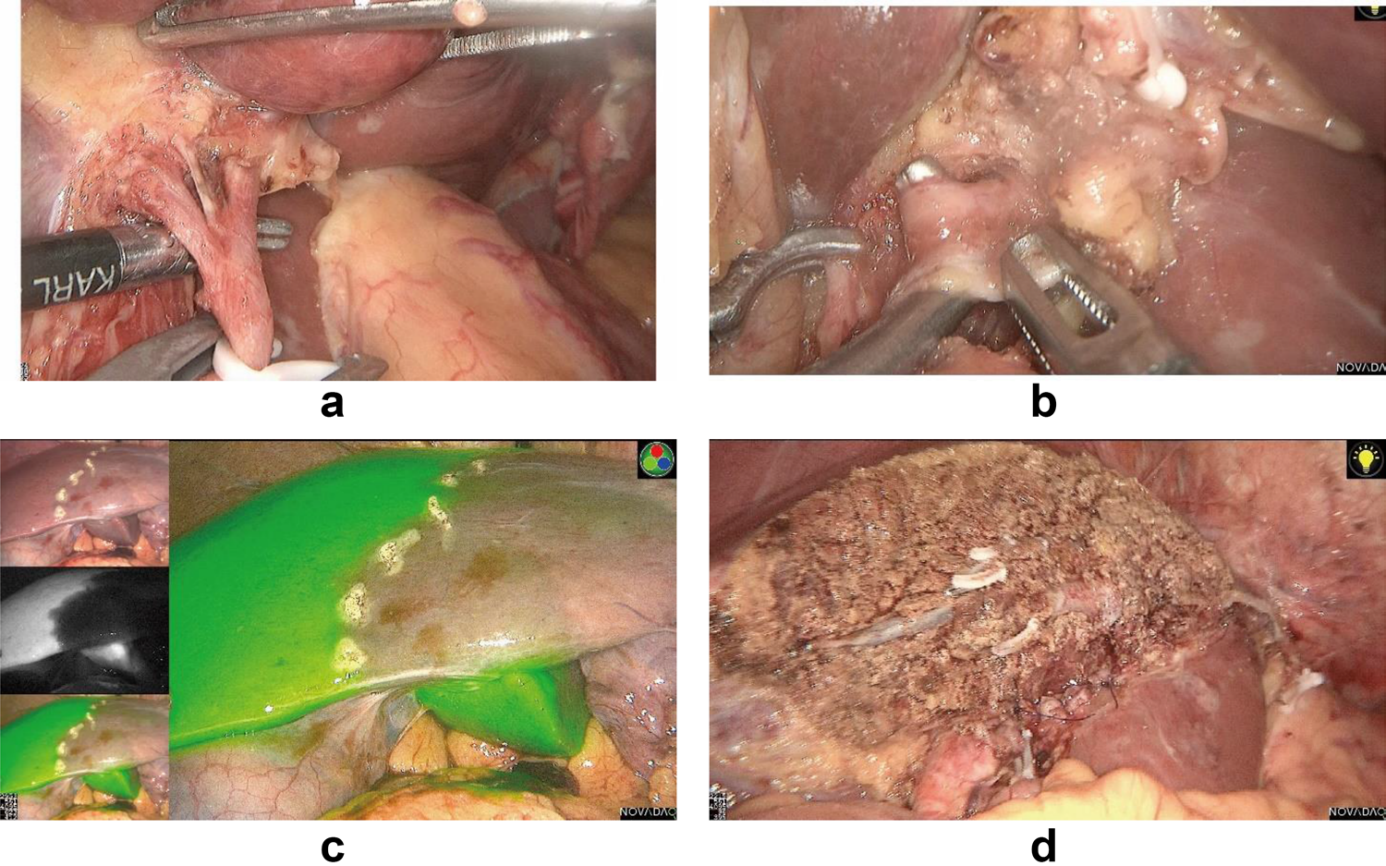
Left hepatic artery (**a**) and portal vein branch (**b**) were dissected and clipped. Then, ICG solution was injected peripheral intravenously. Boundary of hemiliver was apparent on the fluorescence fusion image while the right half stained but the left not (**c**). Exsection plane was marked and left semi-hepatectomy was accomplished (**d**)

ICG dissolved in sterilized water was usually administrated preoperatively via a peripheral vein. ICG distributed in liver tissues with blood flow will be excreted through bile by normal hepatocytes, leading to tumor-specific staining, which enables the tumor to be visualized easily. As fluorescence penetrates the tissue to a depth of only 5–10 mm [6], superficial tumors near the liver capsular can be detected (Fig. 1a). To retain maximal liver parenchyma and reduce the postoperative complications caused by lack of liver volume, parenchymal-sparing non-anatomic resection could be performed, maintaining integrity around the tumors in the liver parenchyma and achieving a curative effect [18]. Intraoperative real-time navigated resection along the fluorescence boundaries was well accepted (Fig. 1b). Anatomic hepatectomy is a better option for non-neoplastic lesions such as hepatolithiasis and tumors located deep in the liver parenchyma not able to be well observed by tumor specific staining. Intraoperatively, the Glissonean pedicle of the lesion-involved segment/lobe was dissected and clipped (Fig. 2a, b), and then ICG was injected intravenously. ICG cannot be delivered into the area to be removed and the remnant liver was stained negatively (Fig. 2c). In the process of liver parenchyma resection, real-time fluorescence navigation was also used to obtain a satisfactory resection section (Fig. 2d). The combination of positive and negative staining can improve the success rate of ICG fluorescence navigation, although extensive clinical training and practical experience is required.

Previous studies have suggested that the timing of preoperative ICG injection may affect the fluorescence imaging of tumor-specific staining. It is thought that the injection should not be given fewer than 4 days preoperatively; and that administration 0–3 days preoperatively may decrease the detection rate of tumors [19, 20]. However, the flexibility of medical resource allocation might be poorer and the waiting time before surgery would be prolonged in this setting. A small retrospective study indicated that a shorter injection time can also result in a satisfactory imaging effect [16]. However, as the ICG administration timing remains inconclusive, another cohort study was begun last year [21]. In our experience, administering ICG 0–3 days preoperatively results in comparable staining and imaging satisfaction to 4–7 days preoperatively (*p* = 0.547). Moreover, there were no significant differences in objective indexes, such as operative time, hilar occlusion time, intraoperative blood loss and postoperative hospital stay (*p* > 0.05 for all), when ICG was administered 0–3 days preoperatively. ICG injection timing closer to the operation day meant shorter preoperative waiting time and better medical resources allocation.

The surgical margin around a malignant tumor is another concern of hepatobiliary surgeons, and related to tumor recurrence and patient survival. R0 resection means that the surgical margin is at least 1 mm away from the tumor boundary and no tumor cells are visible under the microscope. R0 resection was reported to be associated with better long-term survival [22]. ICG fluorescence navigation facilitates the comprehensive application of positive and negative staining and real-time navigation technology to achieve R0 resection [15]. Furthermore, it was demonstrated that narrow resection margin (width ≤ 10 mm) was a predictor for overall survival [23]. A meta-analysis of 34 studies involving 11,147 hepatectomy patients concluded that patients with a wide margin (> 10 mm) had a better prognosis, suggesting that this should be one of the goals of hepatectomy [24]. Recent studies also identified that a wide surgical margin is predictive of a better long-term prognosis [25–27]. During laparoscopic surgery, the lack of tactile perception of laparoscopic forceps and the 2-dimensional image on the monitor in most centers might affect surgeons’ evaluation of the resection range, which could be insufficient to preserve liver parenchyma, resulting in a narrow margin or a positive margin. Our study found no difference in R0 resection rates between the groups, regardless of whether ICG fluorescence navigation was used, but a wide margin was easier to attain in the ICG-FN group. Spearman’s rank correlation and stepwise logistic regression analysis suggested that ICG fluorescence navigation was an important correlation factor and predictor of a wide margin. As a wide surgical margin should be a target of resection of malignant liver tumors [24–27], these results indicated that the patients in the ICG-FN group might have a lower postoperative recurrence rate and longer postoperative survival, although this still needs to be verified by long-term follow-up data.

In conclusion, laparoscopic hepatectomy is now performed widely because of its minimal invasion and enhanced recovery, although it requires skill, training, and practice by surgeons. As an emerging technology, ICG fluorescence navigation is safe and efficient in laparoscopic hepatectomy. It also helps to achieve a wide surgical margin, which often results in a better prognosis. The findings of this study suggest that the administration of ICG closer to the operation, 0–3-days preoperatively, is acceptable.

## Electronic supplementary material

Below is the link to the electronic supplementary material.

Supplementary file1 (DOCX 16 kb)
