# Supplementary material for: Indocyanine green fluorescence navigation in laparoscopic hepatectomy: a retrospective single-center study of 120 cases
Source: Surg Today. 2020 Oct 31;51(5):695–702. doi: 10.1007/s00595-020-02163-8 (PMC8055570; doi:10.1007/s00595-020-02163-8)
Supplement: Supplementary file 1 — Supplementary file1 (DOCX 16 kb) [file 595_2020_2163_MOESM1_ESM.docx]

**Supplemental table 1. Comparison of operative and recovery indices according to anatomical or non-anatomical resection**

|  |  | Anatomical (n=47) |  | Non-anatomical (n=73) |  | *p*-value |
| --- | --- | --- | --- | --- | --- | --- |
| Operative time (min, median [1st/3rd quartiles]) |  | 170 (125-235) |  | 165 (122.5-202.5) |  | 0.325^c^ |
| Hilar occlusion time (min, median [1st/3rd quartiles]) |  | 20 (0-46) |  | 21 (0-49) |  | 0.775^c^ |
| Intraoperative blood loss (ml, median [1st/3rd quartiles]) |  | 100 (50-300) |  | 150 (50-225) |  | 0.815^c^ |
| Postoperative hospital stay (day, median [1st/3rd quartiles]) |  | 8 (6-11) |  | 7 (6-9) |  | 0.221^c^ |

**c: Mann-Whitney U test**

**Supplemental table 2. Comparison of surgical margins according to anatomical or non-anatomical resection**

|  |  | Anatomical (n=25) |  | Non-anatomical (n=53) |  | *p*-value |
| --- | --- | --- | --- | --- | --- | --- |
| Wide surgical margin (n, %) |  | 23 (92%) |  | 42 (79.2%) |  | 0.278^d^ |
| Negative surgical margin (n, %) |  | 24 (96%) |  | 52 (98.1%) |  | 0.541^e^ |

**d: Continuous corrected Chi-square test**

**e: Fisher exact test**
